# The Relation Between Global Longitudinal Strain and Serum Natriuretic Peptide Is More Strict Than That Found Between the Latter and Left Ventricular Ejection Fraction: A Retrospective Study in Chronic Heart Failure

**DOI:** 10.14740/jocmr2370w

**Published:** 2015-10-23

**Authors:** Renato De Vecchis, Cesare Baldi, Giuseppina Di Biase

**Affiliations:** a Cardiology Unit, Presidio Sanitario Intermedio “Elena d’Aosta”, ASL Napoli 1 Centro, Napoli, Italy; b Heart Department, InterventionalCardiology, A.O.U. “San Giovanni di Dio e Ruggi D’Aragona”, Salerno, Italy; c Neurorehabilitation Unit, Clinica “S.Maria del Pozzo”, Somma Vesuviana (NA), Italy

**Keywords:** Chronic heart failure, Natriuretic peptides, Global longitudinal strain, Left ventricular ejection fraction, Speckle tracking

## Abstract

**Background:**

In chronic heart failure (CHF), the finding of elevated levels of the N-terminal fragment of the pro B-type natriuretic peptide (NT-proBNP) is a marker of pathological increase in myocardial ventricular wall stress and detrimental rise in ventricular filling pressures. However, the ensemble of data concerning the relationship between longitudinal deformation indices and NT-proBNP is still rather vague and approximate.

**Methods:**

We carried out a retrospective study that involved 118 patients with CHF admitted to our clinic for CHF outpatients. For inclusion in the study, the CHF patients were required to have undergone at least a determination of global longitudinal strain (GLS) by means of speckle tracking echocardiography and to have practiced at least a determination of NT-proBNP. As regards the two determinations, the one echocardiographic and the other laboratory-based, the former should have been done not more than 24 hours before or after the latter.

**Results:**

Correlation between log (NT-proBNP) and GLS was highly significant (r = 0.8386; P < 0.0001). The observed correlation between log (NT-proBNP) and left ventricular ejection fraction (LVEF) was also significant, but explained a smaller magnitude of the variance (r = -0.5465; P < 0.0001). In multiple linear regression analysis, GLS was shown to be the strongest independent predictor of log (NT-proBNP), within a parsimonious model including age, body mass index, estimated glomerular filtration rate, left atrial volume index, and LVEF (β (regression coefficient) = 305, r_partial_ = 0.7076; P < 0.0001). By using the median value of NT-proBNP (299.5 pg/mL) as a discriminating value for identifying relatively low (i.e., below the median) and relatively high (i.e., above the median) levels of NT-proBNP, GLS was associated with the upper quartiles, whereas LVEF was associated with lower quartiles of NT-proBNP. However, the C statistics for GLS were significantly higher than for LVEF (area under the curve (AUC): 0.949 (GLS) vs. 0.730 (LVEF); P = 0.0030).

**Conclusions:**

In CHF patients, GLS shows a stronger association with NT-proBNP levels with respect to LVEF. Thus, in both CHF with preserved and reduced LVEF, GLS is more accurate compared with LVEF in predicting increased levels of NT-proBNP.

## Introduction

The measurement of the longitudinal strain is used to determine the efficiency of the systolic function of the ventricular myocardial fibers along the longitudinal axis. In this manner, the regional longitudinal as well as the global longitudinal deformation of the myocardium can be evaluated [1-5]. The longitudinal fibers that lie in the sub-endocardial layer are very sensitive to wall stress, and can show abnormal contractile features even in the context of an apparently normal left ventricular ejection fraction (LVEF). Through the systematic measurement of global longitudinal strain (GLS), the strain-based imaging allows us to identify cases of subclinical left ventricular systolic dysfunction attaining a higher sensitivity compared to the traditional technique of the LVEF, assessed by the Simpson’s method [6-8]. However, our knowledge about the relationship between the parameters of echocardiographic strain and activation of natriuretic peptide is still uncompleted [9-13].

### Aims

In order to verify the assumption that neurohormonal activation is more closely related to longitudinal strain compared with LVEF, a retrospective study was performed. In a cohort of outpatients with chronic heart failure (CHF), the already recorded values of N-terminal fragment of the pro B-type natriuretic peptide (NT-proBNP) were collated along with those of LVEF and GLS for identifying the type and strength of any possible pertinent relationship.

## Methods

### Study design

The study was retrospective. It consisted in an examination of a number of medical records of patients with CHF, referred to our centers (E. d’ A. and S.M. d P.) for cardiovascular outpatient investigations from June 2013 to December 2014. As inclusion criteria, the patients were required to suffer from CHF, and to have practiced at least a determination of GLS by means of speckle tracking echocardiography. Furthermore, each patient had done at least a determination of B-type natriuretic peptide, specifically, of NT-proBNP. Moreover, the two determinations, the one echocardiographic and the other laboratory-based, should have been carried out in a condition of substantial chronological simultaneity, that is, the former should have been done not more than 24 h before or after the latter. This close chronological contiguity was made possible by the habitual chronological sequence of the patient’s checking practices in our centers for outpatients with CHF. Indeed, according to the usual diagnostic process, the program for outpatient examinations provided for the temporal concurrence of echocardiographic assessment and determination of natriuretic peptides, that is, these diagnostic practices usually took place on the same day. The study was designed to investigate the possibility of identifying any correlation between GLS, i.e., an echocardiographic parameter, and NT-proBNP, i.e., a lab-based analyte, in each patient. Therefore, it did not aim to calculate retrospectively the impact of GLS or other predictors on one or more clinical outcomes (hospitalizations for heart failure, death from any cause, etc.).

### Echocardiography

The assessment of the images was executed using a Vivid 7 (General Electric, Horten, Norway) echocardiographic machinery. A frame rate of 60 - 75/s was adopted for obtaining the images. Subsequently, they were digitally transferred to a remote workstation for offline analysis (Echopac BT 11.1.0, General Electric, Horten, Norway). All analyses were made by only one experienced operator who had been kept blinded to clinical and biochemical data concerning the patients. Two-dimensional speckle tracking was executed by means of a semiautomatic algorithm. In short, using manual identification, three reference points (two annular and one apical) were selected in each of the three apical views, so as to enable the software to monitor the myocardium in a semi-automated manner in the course of the cardiac cycle. Each ventricular wall was subsequently subdivided into three segments so as to realize the creation of 17 segments covering the whole myocardium. Accurate manual inspection for tracking purposes was performed, and in the case of unsatisfactory tracking, the segment would have been ousted from the analysis. Longitudinal strain curves were built for each segment and the maximum value was determined. The GLS was then computed as the average of all 17 segments.

### Natriuretic peptide determinations

Blood sampling procedures and assay of NT-proBNP from peripheral samples of plasma were gotten after written informed consent within 24 h of echocardiography (either 24 h after or 24 h before echocardiographic examination with GLS determination). Analysis of NT-proBNP was executed using the Modular Analytics E170 NT-proBNP immunoassay (Roche Diagnostics, Mannheim, Germany) immediately after blood sampling.

### Statistical analysis

All statistical tests were performed with a commercially available statistical analysis program (SPSS 15.0 for Windows, SPSS Inc., Chicago, IL, USA). The distribution of the data was assessed using the one-sample D’Agostino-Pearson test. Continuous variables displaying normal distribution were expressed as mean ± SD, while values with asymmetric distribution were expressed as medians with interquartile ranges. Categorical variables were presented as %. The comparisons were made by means of Student’s *t*-test or ANOVA (for continuous variables) or by applying the Chi-square test (categorical variables).

A general linear regression model was built by including into it both continuous and categorical variables. NT-proBNP was logarithmically converted (log10) to make the variance as stable as possible according to the assumptions of multiple regression. A P-value < 0.05 was regarded significant. All relevant variables were entered and a parsimonious model was realized by subsequent variable elimination in a backward stepwise manner using P < 0.1 as a retention criterion.

Values of NT-proBNP above the median (50th percentile) were compared with those located below the median (lower quartiles) and separate logistic regression models were set up for GLS and LVEF as a single predictor variable. Comparison of each method’s predictive potential was performed by comparing the C-statistic derived from the area under the receiver operating characteristic (ROC) curves. Statistical results were regarded significant when P < 0.05 (two-sided). In our laboratory for speckle tracking echocardiography, in the course of the year 2014, the intra-observer variability of the GLS measurements had been assessed by calculating intra-observer variations in 20 successive subjects (“Results”).

## Results

**Figure 1:**
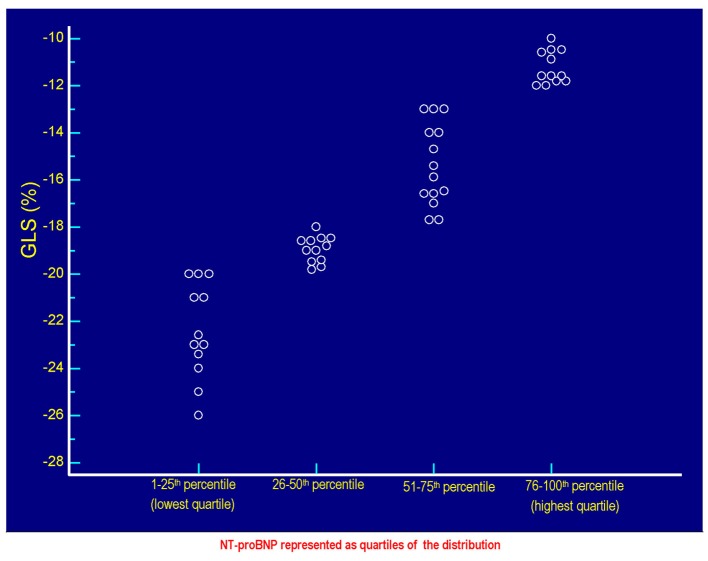
The values of GLS (%) of each patient are plotted against the respective values of serum NT-proBNP. Each circlet, corresponding to a given value of GLS, falls in one of the four groups of circlets coincident with the quartiles of NT-proBNP derived from the categorization of the entire range of possible NT-proBNP values in the context of the 50 patients included in the study. The lowest quartile includes the NT-proBNP values ≤ 58 pg/mL, the quartile between the 26th percentile and the median (50th percentile) includes values > 58 and ≤ 299.5 pg/mL, the quartile between 51th and the 75th percentile includes the values > 299.5 and ≤ 1,405 pg/mL, the highest quartile incorporates the values > 1,405 pg/mL up to the highest value found, i.e., 4,123 pg/mL. Note that the less pronounced cases of global longitudinal systolic deformation are grouped in the highest quartile of the distribution of the values of NT-proBNP, while the most efficient cases of GLS (characterized by the most negative values) are grouped in the lowest quartile of NT-proBNP. Please note also that the increasing (i.e., progressively less negative) values of GLS are coupled with progressively increasing values of NT-proBNP, according to a direct linear relationship (Fig. 2). GLS: global longitudinal strain; NT-proBNP: N-terminal fragment of the prohormone of the B-type natriuretic peptide.

**Figure 2:**
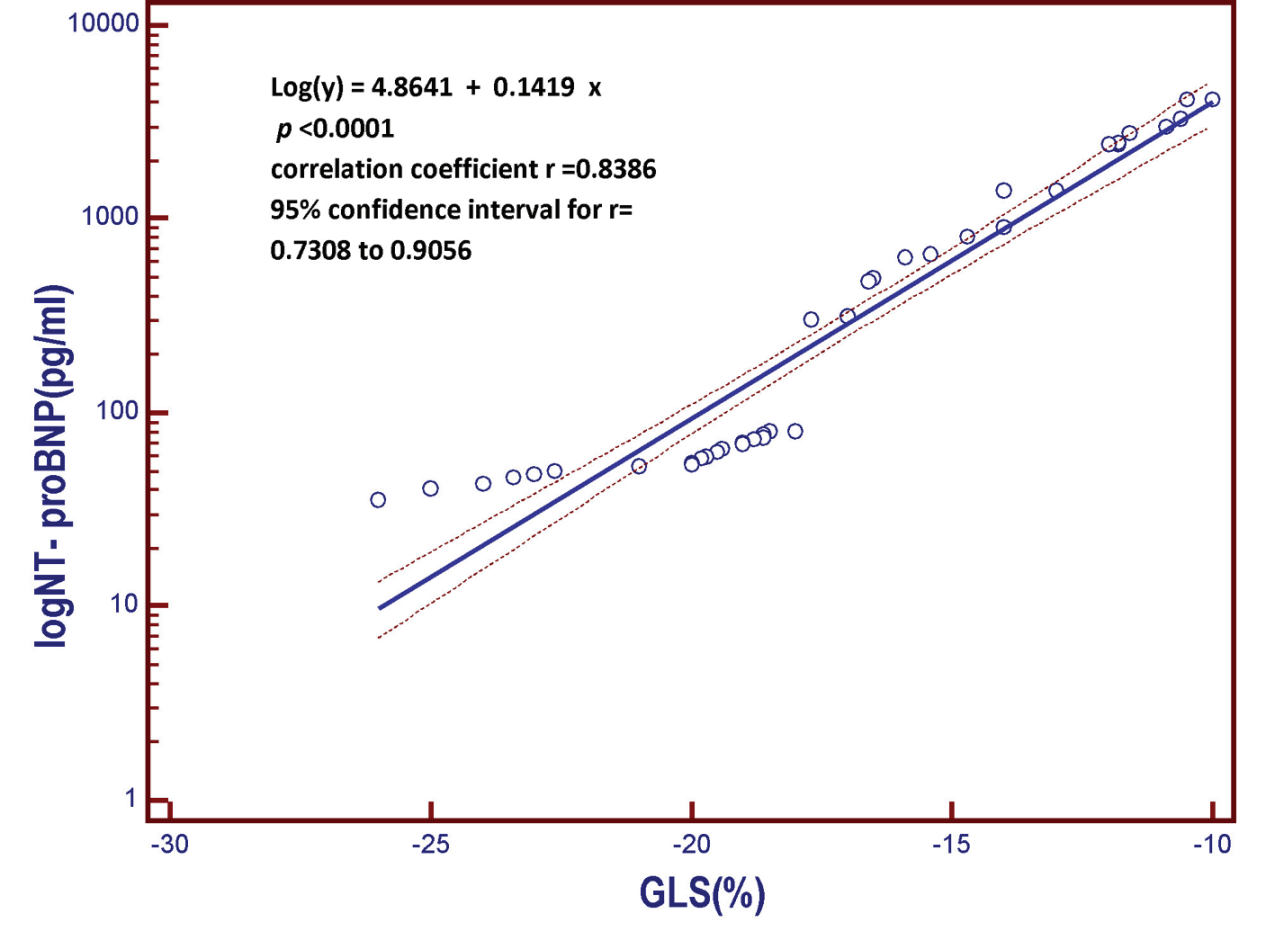
Linear regression model derived from the patient population of the present study. The model includes the B-type natriuretic peptide (log NT-proBNP), which is the dependent variable y, on the vertical axis, and the global longitudinal strain (GLS) on the horizontal axis. As the global longitudinal systolic deformation of the left ventricular chamber is reduced, the value of the NT-proBNP, a marker of myocardial left ventricular intracavitary tension, progressively increases. In other words, those who have the less negative values of global systolic longitudinal strain, that is, who have weaker systolic shortening of the left ventricular chamber due to less valid longitudinal contraction of the subendocardial fibers, are also those having the highest circulating levels of B-type natriuretic peptide. More impaired is the longitudinal contractile function, the higher the value of the serum natriuretic peptide. GLS: global longitudinal strain; NT-proBNP: N-terminal fragment of the pro B-type natriuretic peptide; pg: picograms; log: logarithmic transformation of Y.

**Figure 3:**
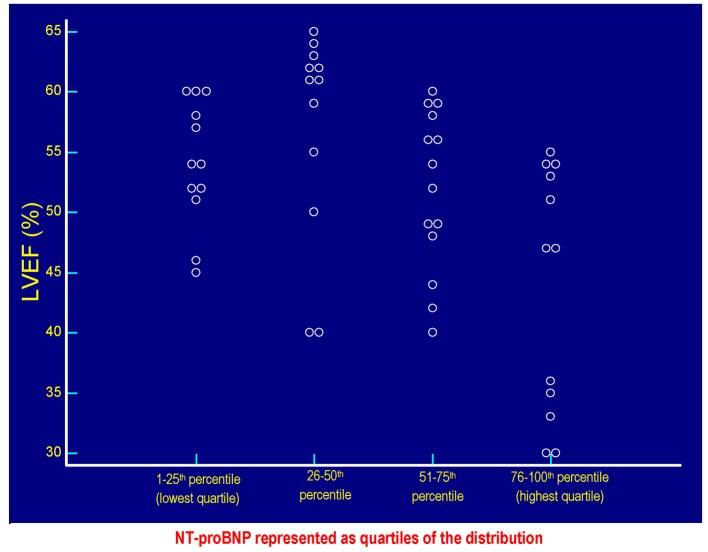
The values of LVEF (%) of each patient are plotted against the respective values of serum NT-proBNP. Each circlet, corresponding to a given value of LVEF, falls in one of the four groups of circlets coincident with the quartiles of NT-proBNP derived from the categorization of the entire range of possible NT-proBNP values in the context of the 50 patients included in the study. The lowest quartile includes the NT-proBNP values ≤ 58 pg/mL, the quartile between the 26th percentile and the median (50th percentile) includes values > 58 and ≤ 299.5 pg/mL, the quartile between 51th and the 75th percentile includes the values > 299.5 and ≤ 1,405 pg/mL, the highest quartile incorporates the values > 1,405 pg/mL up to the highest value found, i.e., 4,123 pg/mL. Please note that the values of LVEF are distributed within the whole range of the possible NT-proBNP values, so it is not possible to identify the existence of a significant inverse linear relationship between LVEF and serum NT-proBNP in the study population (Fig. 4). LVEF: left ventricular ejection fraction; NT-proBNP: N-terminal fragment of the prohormone of the B-type natriuretic peptide.

**Figure 4:**
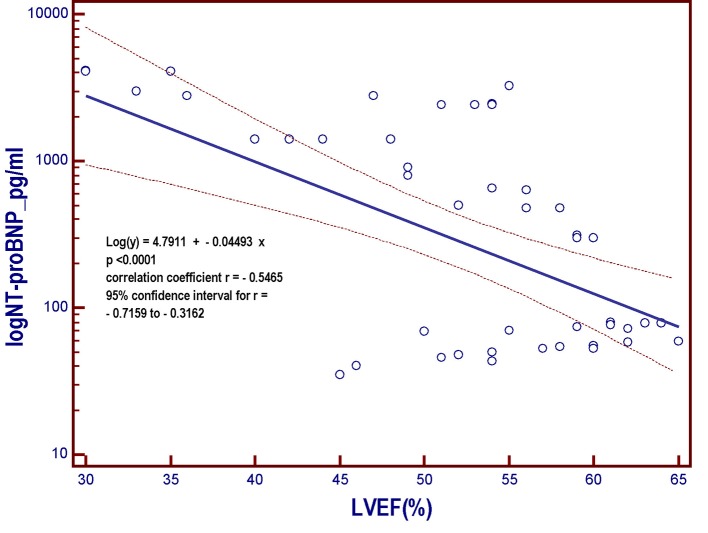
Linear regression model derived from the patient population of the present study. The model includes the NT-proBNP that undergoes logarithmic transformation (log NT-proBNP), assumed as the dependent variable y and placed on the vertical axis, and the left ventricular ejection fraction (LVEF), put on the horizontal axis. As the LVEF progressively decreases across the whole range of the possible values that it can assume within the entire patient population, the value of the NT-proBNP, a marker of myocardial left ventricular intracavitary tension, progressively increases. However, the strength of the relationship is much less marked (r = -0.5465) with respect to that characterizing the relation between log NT-proBNP and GLS (r = 0.8386; Fig. 2). NT-proBNP: N-terminal fragment of the pro B-type natriuretic peptide; pg: picograms; LVEF: left ventricular ejection fraction; log: logarithmic transformation of Y; GLS: global longitudinal strain.

The total study population consisted of 118 patients all suffering from CHF, who were cared for in our clinic for heart failure outpatients. Twenty-six patients were excluded from the analysis due to atrial fibrillation (23 patients) and ventricular paced rhythm (three patients). Forty-two patients were excluded due to poor image quality causing three or more myocardial segments to be incorrectly traced by the speckle tracking algorithm. Thus, 50 patients were included in the analyses (median age 69 years (interquartile range 56 - 78 years), 32 (64%) males). Mean LVEF was 52% and mean LV GLS was -15%. The level of NT-proBNP ranged from 35 to 4,123 pg/mL, with a median of 299.5 pg/mL (interquartile range 58 - 1,405 pg/mL). The overall linear relationships between log (NT-proBNP) and GLS and LVEF are shown in Figures 1-4. Correlation between log (NT-proBNP) and GLS was significant (P < 0.0001, r = 0.8386) (Fig. 2). The observed correlation between log (NT-proBNP) and LVEF was also significant, but explained a smaller magnitude of the variance (P < 0.0001, r = -0.5465) (Fig. 4). In CHF patients with reduced ejection fraction (HFREF) (17 patients, 34%) and in those with preserved ejection fraction (HFpEF) (33 patients, 66%) analyzed separately, log (NT-proBNP) exhibited a stronger overall correlation with GLS (HFREF, P < 0.0001, r = 0.8426; HFpEF, P < 0.0001, r = 0.8843) compared with LVEF (HFREF, P = 0.1568, r = -0.3592; HFpEF, P = 0.0502, r = -0.3437). In multiple linear regression analysis, GLS was shown to be as an independent predictor of log (NT-proBNP), within a parsimonious model including age, body mass index, estimated glomerular filtration rate, left atrial volume index, and LVEF (β (regression coefficient) = 305, r_partial_ = 0.7076; P < 0.0001) (Table 1).

**Table 1:** The Multiple Linear Regression Analysis Shows That GLS Is the Best Independent Predictor (rpartial = 0.7076) of High Levels of NT-proBNP, Within a Parsimonious Model Including Age, BMI, eGFR, LAVi, and LVEF

Dependent variable y	NT-proBNP (pg/mL)				
Sample size	50				
Coefficient of determination R^2^	0.8539				
R^2^-adjusted	0.8335				
Multiple correlation coefficient	0.9240				
Residual standard deviation	521.4549				
Regression equation					
Independent variables	Coefficient	Std. error	r_partial_	t	P
Constant	11,132.85				
Age	4.9594	6.4871	0.1158	0.76	0.4487
BMI	21.0525	26.0587	0.1223	0.80	0.4236
eGFR	-1.5465	3.5382	-0.06651	-0.44	0.6642
GLS (%)	305.0159	46.4509	0.7076	6.56	< 0.0001
LAVi (mL/m^2^)	-97.8632	47.3994	-0.3003	-2.06	0.045
LVEF (%)	-54.7913	11.4641	-0.589	-4.8	< 0.0001
					

Analysis of variance					
F-ratio	41,873				
Significance level	P < 0.0001				

BMI: body mass index; eGFR: estimated glomerular filtration rate; GLS: global longitudinal strain; LAVi: left atrial volume index; LVEF: left ventricular ejection fraction; r: correlation coefficient.

Univariate logistic regression analysis was performed separately for GLS and LVEF, by using the median value of NT-proBNP (namely 299.5 pg/mL) as a discriminating value for identifying relatively low (i.e., below the median) and relatively high (i.e., placed above the median) levels of NT-proBNP. In this manner, using univariate regression logistic analysis, in the overall study population, GLS was associated with significantly increased probability (odds ratio (OR) = 1.4; P < 0.0001; 95% CI = 1.30 - 1.51) of the highest levels of NT-proBNP (i.e., > 299.5 pg/mL). Similarly, using univariate regression logistic analysis, LVEF was associated with significantly increased probability (OR = 0.92; P < 0.0001; 95% CI = 0.90 - 0.94) of reduced levels of NT-proBNP (i.e., ≤ 299.5 pg/mL).

**Figure 5:**
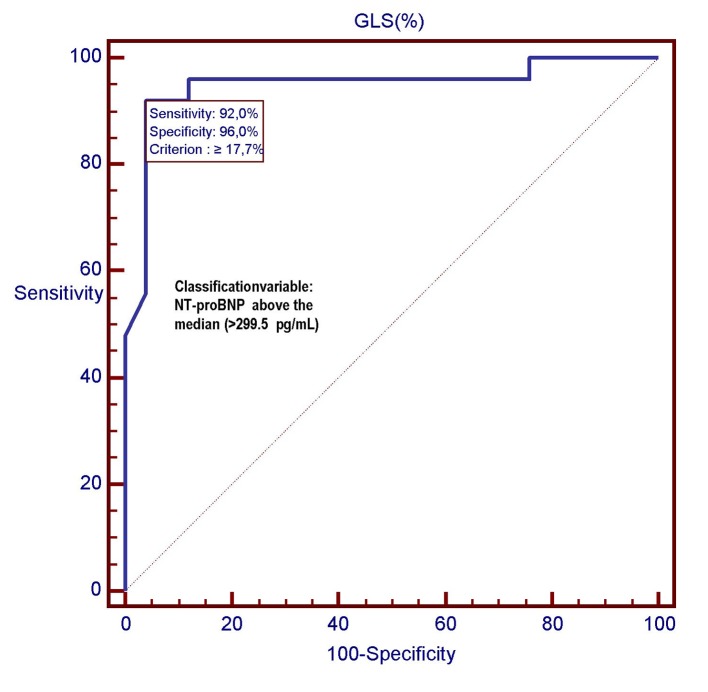
In this ROC plot, there is the representation of the diagnostic performance (AUC = 0.949) of GLS as a predictor of relatively high values (> 299.5 pg/mL) of NT-proBNP among 50 CHF patients. GLS: global longitudinal strain; ROC: receiver operating characteristic; NT-proBNP: N-terminal fragment of the pro B-type natriuretic peptide; pg: pictograms.

**Figure 6:**
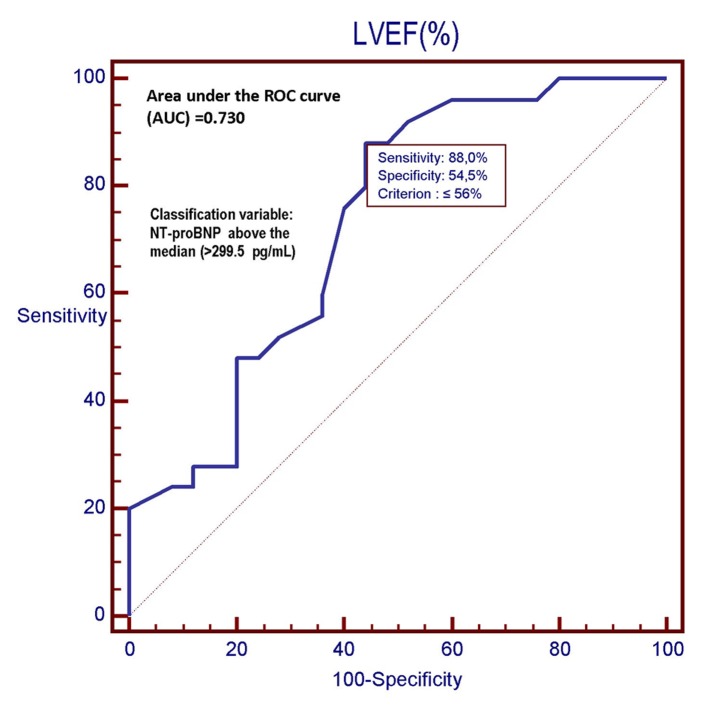
In this ROC plot, there is the representation of the diagnostic performance (AUC = 0.730) of the LVEF as a predictor of relatively high values (> 299.5 pg/mL) of NT-proBNP among 50 CHF patients. LVEF: left ventricular ejection fraction; ROC: receiver operating characteristic; NT-proBNP: N-terminal fragment of the pro B-type natriuretic peptide; pg: picograms.

**Figure 7:**
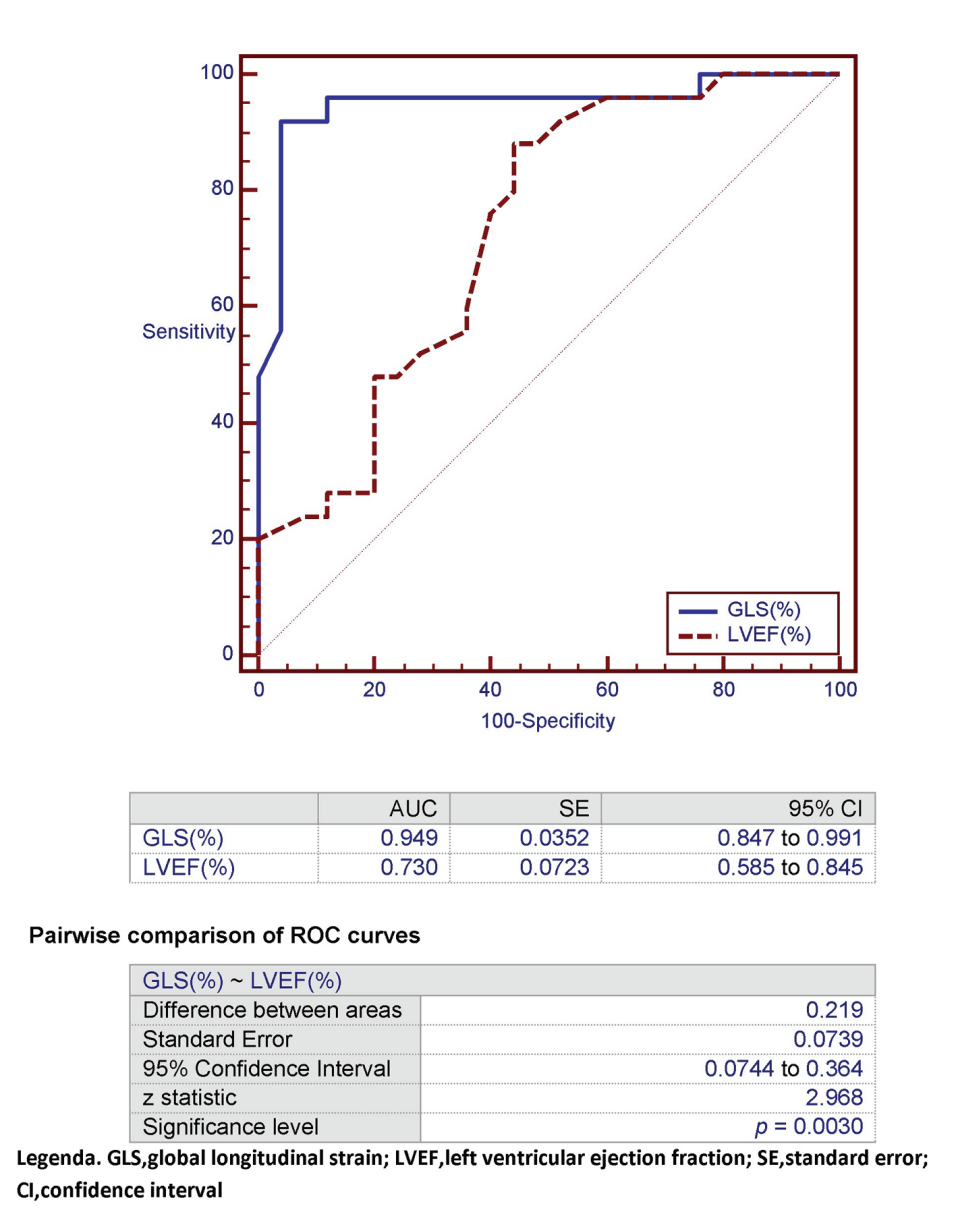
In this ROC plot, the area under the curve (AUC) that characterizes GLS for detection of an NT-proBNP > 299.5 pg/mL (classification variable) is compared with the corresponding AUC exhibited by LVEF within the study population. GLS: global longitudinal strain; LVEF: left ventricular ejection fraction; SE: standard error; CI: confidence interval.

However, the C-statistics for GLS were significantly higher than for LVEF (AUC: 0.949 (GLS) vs. 0.730 (LVEF); P = 0.0030) (Fig. 5-7). In other words, the ability of GLS to predict the association with a relatively high level of NT-proBNP, i.e., located above its 50th percentile, was compared with that exhibited by LVEF. In this way, the value of GLS ≥ -17.7% was shown to have the best diagnostic accuracy (sensitivity = 92%; specificity = 96%; positive likelihood ratio (+LR) = 23; negative likelihood ratio (-LR) = 0.083) in predicting the presence of relatively high (i.e., above the median) levels of NT-proBNP, with an AUC of 0.949. Similarly, among all values of LVEF detected in the patient population, an LVEF ≤ 56% could achieve the best diagnostic accuracy (sensitivity = 88%; specificity = 56 %; +LR = 2; -LR = 0.21) in the detection of relatively high levels (i.e., above the median) of NT-proBNP, although showing an AUC of 0.730, namely, significantly lower than that exhibited by GLS (P = 0.0030) (Fig. 7). As already specified in the previous section (“Echocardiography”), all echocardiographic measurements concerning the strain were made by only one experienced operator who per customary practice was almost always kept blinded to clinical and biochemical data concerning the patients. For this experienced sonographer, Bland-Altman analysis demonstrated a good intra-observer agreement with a small non-significant bias for GLS. In fact, mean difference ± 2 standard deviations for GLS was 0.30±0.7% with a percentage of error of 3.1% calculated from the repeated examination of 20 successive individuals.

## Discussion

The relationship between GLS and B-type natriuretic peptide has been described by various authors [13-18], in particular by Ersboll, in several series comprising patients affected by acute myocardial infarction [16-18]. Some previous works agree with our research with respect to the point that GLS is a more reliable predictor of levels of B-type natriuretic peptide than the LVEF [16, 19]. The previous studies, as in our case, have been conducted with the use of the amino-terminal fragment of B-type natriuretic peptide. In fact, it seems that the systolic impairment and dysfunction of the longitudinal fibers of the left ventricle in the subendocardial layers, when greatly pronounced, very frequently entail an end-diastolic pressure increase of the left ventricle which results in a stimulus to the synthesis and secretion of B-type natriuretic peptide. LVEF would describe with less fidelity and in a more approximate manner the increased load of work stress of the fibers of the inner layers of the left ventricle [16]; therefore, its relationship with this biochemical index of end-diastolic pressure would be less strict compared to GLS. Moreover, in our study, in a large subgroup of patients characterized by preserved LVEF, the relationship between this parameter and the levels of NT-proBNP was relatively weak, while GLS values continued to show a clear linear direct relationship with log (NT-proBNP). In another study that dealt with the speed of the shortening of the longitudinal fibers surrounding the mitral orifice (s’ velocity, measured by tissue Doppler imaging (TDI)), an inverse linear relationship was noted between serum levels of BNP and systolic shortening velocity at the level of the longitudinal periorificial mitral fibers [12].

Thus, in CHF, depressed longitudinal function as expressed by annular velocity is related to NT-proBNP levels. In this regard, in our study, we found significantly stronger correlation between GLS and NT-proBNP compared with that detected between the annular velocity s’ and NT-proBNP in a previous study [20]. We infer that these findings reflect that s’ to a lesser extent than GLS mirrors the heterogeneous contraction associated with CHF. Furthermore, even a more recent study demonstrated that deformation indices rather than TDI-derived myocardial velocities identified systolic dysfunction in HFpEF patients [21].

Biplane LVEF calculation utilizing the Simpson method is dependent on visual display of the endocardial margin, which can be very difficult in patients with poor acoustic window. This limitation could concur to the superiority of GLS over LVEF. However, similar difficulties, though to a lesser extent, are present when performing two-dimensional speckle tracking [22].

A major determinant of NT-proBNP in patients with CHF is believed to be wall stress (WS), which depends on the LV diastolic filling pressure, wall thickness, and LV diastolic diameter. Thus, in the presence of increasing filling pressures, given a constant wall thickness in the absence of compensatory concentric hypertrophy, the WS will rise.

An elevated E/e’ ratio and shortened mitral valve deceleration time provide prognostic information in patients with heart failure and mirror increasingly restrictive filling, high filling pressure, and increased WS [23].

The myocardial anatomy consists of sub-endocardial fibers longitudinally directed with an oblique array, whereas mid-wall fibers are arranged according to a circumferential disposal. This entails a non-uniform distribution of WS, with decreasing values from endo- to epicardium. The sub-endocardial longitudinal fibers have a less pronounced curvature compared to the mid-wall circumferential fibers, so, according to the Laplace’s law that rules a three-dimensional shell, the WS will be higher at the level of the sub-endocardial longitudinal fibers [24].

In daily practice, myocardial contractility is based on LVEF by echocardiography using the Simpson biplane model. However, LVEF lacks sensitivity to identify accurately myocardial contractility impairment, which first affects sub-endocardial layers and longitudinal component. In addition, LVEF is influenced by load conditions and depends on the experience of the operator [25]. In the present study, we demonstrated that in patients with CHF, the association that relates GLS (assessed by speckle tracking) to NT-proBNP is stronger with respect to the linear inverse relationship that unites the latter to LVEF.

The relation between longitudinal function and WS has not been investigated in detail with invasive hemodynamic monitoring in humans. However, an animal study by Donal et al [26] plainly showed that longitudinal wall kinetics was more vulnerable to increased WS, whereas a preserved circumferential function was able to leave unaltered the radial fractional shortening of the chamber diameter.

### Study limitations

Relatively exiguous sample size and retrospective design are the main limitations of our study. Another limitation is the lack of comparison with the TDI-derived indexes, i.e., both systolic and diastolic mitral annular velocities, which prevented us from using the E/e’ in our analyses and comparisons. However, a comparative evaluation of deformation indices vs. TDI-derived systolic velocities will be the subject of a forthcoming study to be carried out by our research team.

### Conclusions

Impaired values (i.e., less negative) of LV GLS are associated with increased plasma concentrations of B-type natriuretic peptide. Our data suggest that left ventricular myocardial mechanics estimated by LV GLS mirrors myocardial WS more accurately with respect to LVEF. In fact, GLS is superior to LVEF in the prediction of elevated levels of NT-proBNP, which in turn have to be regarded as the immediate and almost unavoidable epiphenomenon of the increased intracavitary tension, related to cardiac overload.
